# Supplementary material for: Brachypodium Antifreeze Protein Gene Products Inhibit Ice Recrystallisation, Attenuate Ice Nucleation, and Reduce Immune Response
Source: Plants (Basel). 2022 May 31;11(11):1475. doi: 10.3390/plants11111475 (PMC9182837; doi:10.3390/plants11111475)
Supplement: Supplementary file 1 [file plants-11-01475-s001.zip › File S3.pdf]

plants

Brachypodium Antifreeze Protein Gene Products Inhibit Ice Recrystallisation, Attenuate Ice Nucleation, and Reduce Immune Response

Collin L. Juurakko [1,*], George C. diCenzo [1] and Virginia K. Walker [1,2]

[1] Department of Biology, Queen's University, Kingston, ON K7L 3N6, Canada; george.dicenzo@queensu.ca (G.C.D.); walkervk@queensu.ca (V.K.W.)
[2] Department of Biomedical and Molecular Sciences, School of Environmental Studies, Queen's University, Kingston, ON K7L 3N6, Canada
* Correspondence: 11cj10@queensu.ca; Tel.: +1-613-533-6000 (ext. 77360)

Supplemental Tables

Table S1. Ice nucleation assays with mean differences in T_{90} values (the temperature when 90% of samples nucleate) for ice nucleating proteins (INPs; 50 µg mL^{-1}) in combination with cold-acclimated (CA) and non-acclimated (NA) wildtype *Brachypodium distachyon* lysates (*Bd*) and transgenic knockdown lines (prOmiRBdIRI-1e and prOmiRBdIRI-3c), or with tannic acid (TA; 100 mM), TA and CA-treated *Bd,* or with INPs pre-incubated at 37 °C for 24 h (heat) compared to INPs alone; in all cases, values shown are the mean of three replicates with standard deviation. All experiments included buffer controls that did not nucleate at the temperatures used (not shown).

Sample	ΔT_{90} (°C)
INP + *Bd* (CA)	−1.55 ± 0.28
INP + *Bd* (NA)	0.00 ± 0.59
INP + prOmiRBdIRI-1e (CA)	0.52 ± 0.32
INP + prOmiRBdIRI-1e (NA)	−0.04 ± 0.04
INP + prOmiRBdIRI-3c (CA)	−0.03 ± 0.37
INP + prOmiRBdIRI-3c (NA)	0.10 ± 0.24
INP + tannic acid	−2.28 ± 0.34
INP + tannic acid + *Bd* (CA)	−2.88 ± 0.54
INP + Heat	−3.33 ± 1.17

Table S2. Theoretical interface areas (Å^2) from the PDBePISA interface output between representative receptor from the LRR translation product of *BdIRI1* (LRR1), *BdIRI3* (LRR3) and *BdIRI4* (LRR4) and various flg22-α or flg22-γ ligand peptide chains, as well as the difference in total solvation energies of isolated and complexed structures where negative values indicate hydrophobic interfaces, or positive protein affinity, not including hydrogen bonds across interfaces (shown as Δ^iG kcal mol^{-1}), in addition to the observed solvation free energy gain where $p < 0.5$ indicates interfaces with higher than average hydrophobicity for the given structure suggesting the surface is interaction-specific (the Δ^iG *p*-value).

Protein (Receptor)	Peptide (Ligand) *	Interface Area (Å^2)	Δ^iG kcal mol^{-1}	Δ^iG *p*-Value
LRR1	Gamma	568.5	−3.6	0.437
LRR1	Alpha	232.8	−2.1	0.415
LRR1	GammaP#1	115.1	−0.3	0.600
LRR1	GammaP#2	54.6	0.0	0.544
LRR1	GammaP#3	168.5	−0.6	0.415
LRR1	GammaP#4	291.4	−0.8	0.441
LRR1	GammaP#5	198.8	0.1	0.433
LRR3	Gamma	501.6	−7.2	0.225
LRR4	Gamma	467.3	−6.8	0.243
LRR3 #	LRR4 #	1057.5	−12.9	0.206
LRR3	LRR4	675.0	−8.4	0.266
LRR3 + 4	Gamma	567.9	−7.3	0.207
LRR3 #	LRR3 #	853.4	−12.4	0.161
LRR3	LRR3	588.1	−8.8	0.211
LRR3 ×2	Gamma	757.1	−9.1	0.286
LRR4 #	LRR4 #	674.1	−6.5	0.401
LRR4	LRR4	887.7	−7.4	0.371
LRR4 ×2	Gamma	1559.2	−11.9	0.369
LRR3 ×4	Gamma	1224.7	−6.7	0.516
*Bd*RLP23	Gamma	673.0	−1.1	0.603
*Bd*RLP23	Alpha	268.2	−1.0	0.544
*Bd*PEPR1	Gamma	713.1	−1.5	0.592
*Bd*PEPR1	Alpha	602.8	1.1	0.767
*At*FLS2	Gamma	912.0	−6.4	0.373
*At*FLS2	flg22 $	952.5	−8.4	0.317
*At*FLS2	Alpha	231.6	−0.0	0.655
*Bd*FLS2	Gamma	588.0	−6.2	0.227
*Bd*FLS2	Alpha	238.2	−2.8	0.369

* GammaP# refer to random permutations of the flg22-y peptide generated by the shuffle protein tool (https://www.bioinformatics.org/sms2/shuffle_protein.html (accessed on November 1 2021)). # Denotes the LRR dimer folded independently without the flg22 peptide in the complex. $ Denotes the flg22 peptide from the crystal structure was used (QRLSTGSRINSAKDDAAGLQIA).

Table S3. Primer sequences used for Gibson assembly cloning of LRR constructs in pCambia1305.1 for transient expression of *Bd*LRRs in *Arabidopsis*. Lower case letters indicate the 20 bp overhang portion overlapping the plasmid and upper-case letters indicate the portion of primer binding to the gene sequence with melting temperature and GC content indicated for only the binding portion of the primer.

Primer	Sequence	Tm (°C)	GC%
BdLRR1-F	5′-gaacacgggggactcttgaccATGGCAAAATGCTGGCTGCC-3′	60.3	55%
BdLRR1-R	5′-aaatttaccctcagatctacCCCAGTTATTGTGTTTGGTT-3′	53.1	40%
BdLRR3-F	5′-gaacacgggggactcttgaccATGGCAAAATACTGGCTGCT-3′	55.6	45%
BdLRR3-R	5′-aaatttaccctcagatctacCCCAGTTATTGTATTTGGTT-3′	49.8	35%
BdLRR7-F	5′-gaacacgggggactcttgaccATGGCAAAATGCTGGCTGCT-3′	58.7	50%
BdLRR7-R	5′-aaatttaccctcagatctacCCCAGTTATTGTATTTGGTT-3′	49.8	35%

Supplemental Figures

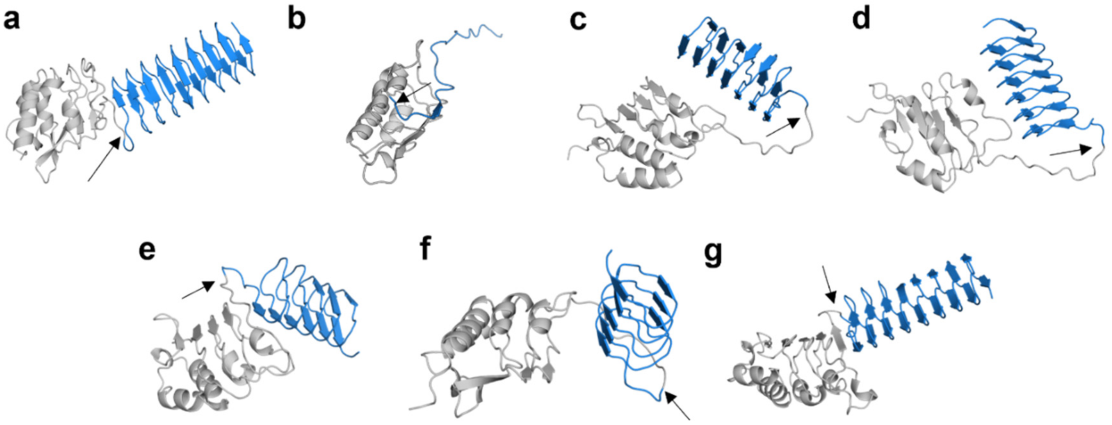

Figure S1. AlphaFold protein models of the 7 full *BdIRI* primary translation products with leucine-rich repeat (LRR) domains in grey and antifreeze protein (AFP) domains in blue; the hydrolytic cleavage site is located between the two. (**a**) *BdIRI1*. (**b**) *BdIRI2* (shown as only having a LRR domain without the AFP protein but the mutation producing this truncated primary translation product is not found in other *Brachypodium* accessions, where an AFP similar to the other *BdIRI* genes is found). (**c**) *BdIRI3*; (**d**) *BdIRI4*; (**e**) *BdIRI5*; (**f**) *Bd*IRI6; (**g**) *Bd*IRI7. Arrows indicate linker region between the LRR and AFP with the endopeptidase cleavage site occurring at the colour change between highlighted structures.

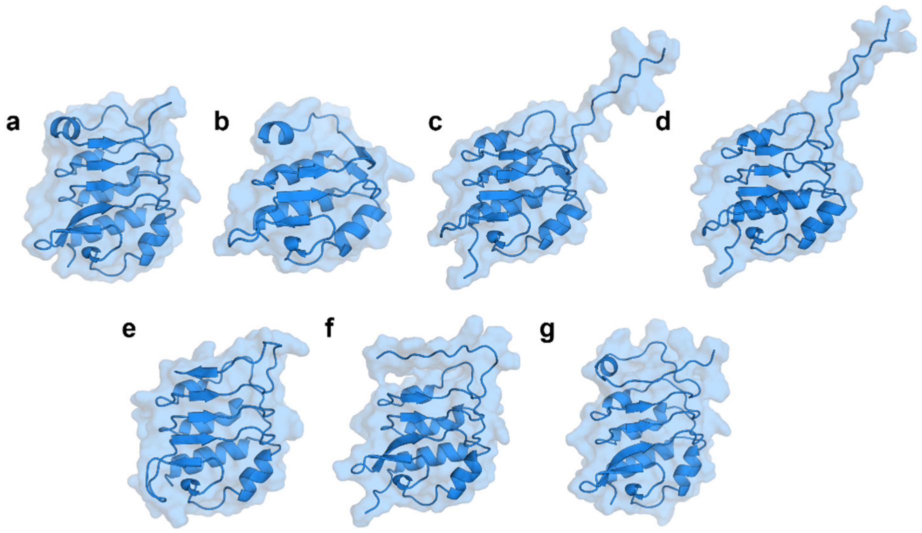

Figure S2. AlphaFold models of *BdIRI* leucine-rich repeat (LRR) domains folded independently and showing the solvent-accessible surface area. (**a**) LRR1; (**b**) LRR2; (**c**) LRR3; (**d**) LRR4; (**e**) LRR5; (**f**) LRR6; (**g**) LRR7.

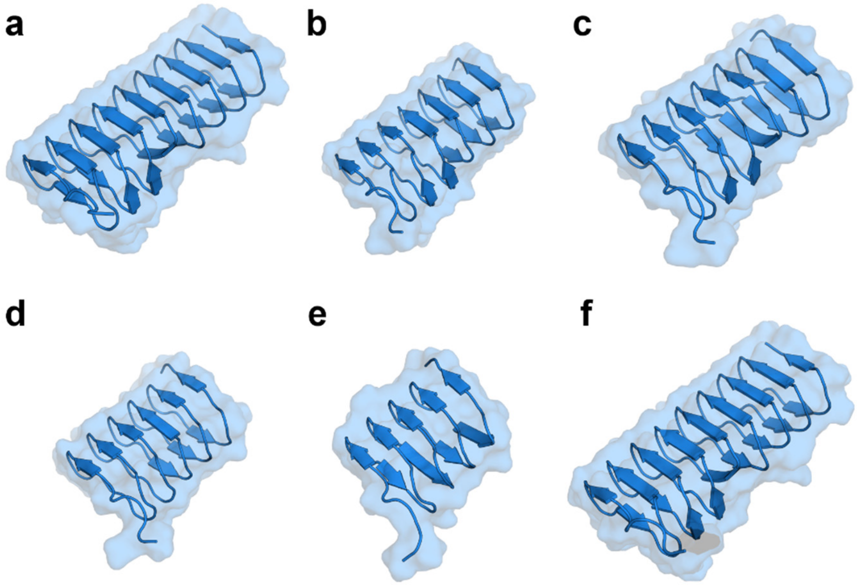

Figure S3. AlphaFold models of *BdIRI* gene antifreeze protein (AFP) domains folded independently and showing the solvent-accessible surface area. (**a**) *Bd*AFP1; (**b**) *Bd*AFP3; (**c**) *Bd*AFP4; (**d**) *Bd*AFP5; (**e**) *Bd*AFP6; (**f**) *Bd*AFP7.

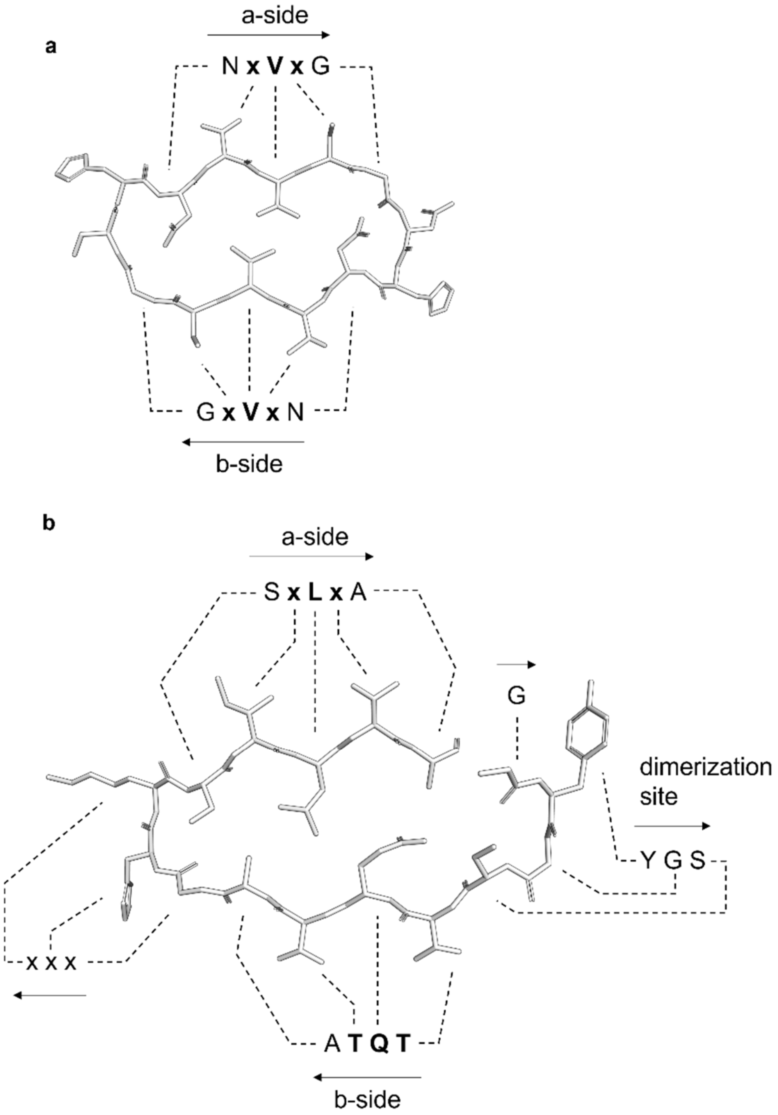

Figure S4. Stick representations of ice-binding motif repeats of AFPs and INPs. (**a**) *Brachypodium distachyon* AFP repetitive "a and b ice-binding faces" on the top and bottom, respectively, of the cross-sectional depiction of the sequence N**x**V**x**G, where x is an outward-facing, hydrophilic residue and ice-binding conserved triplets are indicated in bold. (**b**) A single representative *Pseudomonas syringae* INP water-organising tandem GYGS**TQT**AxxxS**x**L**x**A repeat, where x is an outward-facing hydrophilic residue and the "a and b water-organising faces" on the top and bottom, respectively, are shown in bold, with the conserved sequence forming the tyrosine ladder also shown. Residues are labelled with dashed lines and arrows indicate amino- to carboxyl-sequence directionality corresponding to the model.

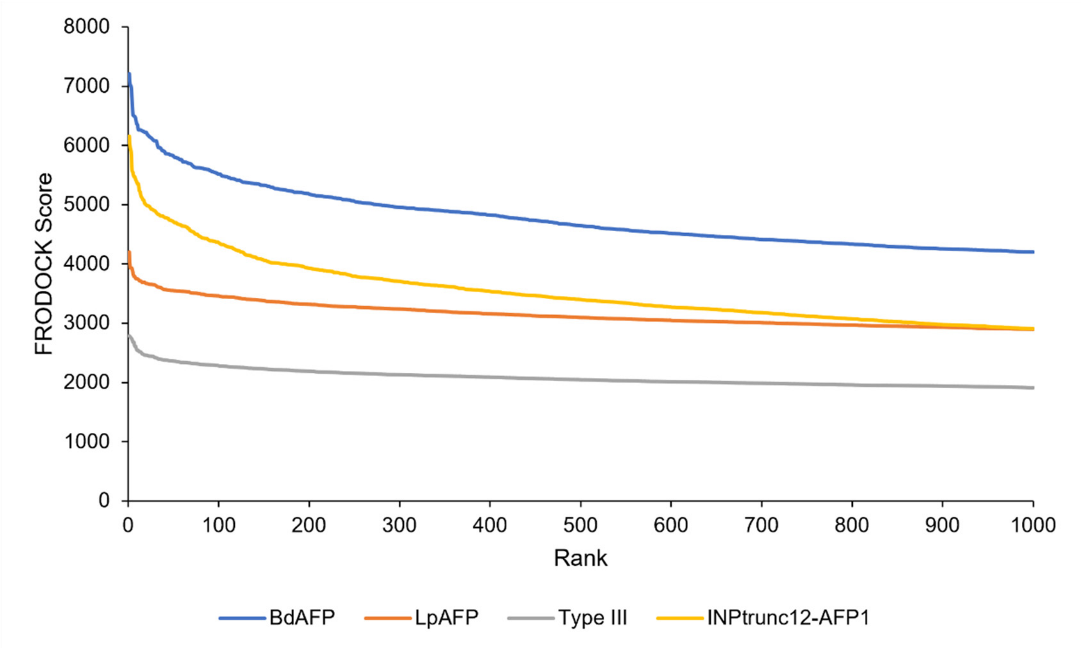

INP	AFP	Max Score (1st)	10th Score	AFP Face	INP Face	Min Score (1000th)
**Full INP**	**BdAFP**	7212	6349	100% a	100% a	4199
**Full INP**	**LpAFP**	4203	3747	80% b	80% a	2895
**Full INP**	**Type III AFP**	2783	2538	50% yes	100% a	2538
**INP Trunc 12**	**BdAFP**	6159	5354	100% a	100% a	2904

Figure S5. Summaries of FRODOCK correlation scores and rankings for predicted docking of *Brachypodium distachyon* antifreeze protein (*Bd*AFP), *Lolium perenne* AFP (*Lp*AFP), and a Type III AFP from the fish, *Macrozoarces americanus*, against *Pseudomonas syringae* ice nucleating protein, INP, using the InaZ variant as a model as well as truncated INPs using a segment of the beta-solenoid containing 12 of the tandem repeats modelled with *Bd*AFP. The top 1000 rankings and scores are shown (**top**) and the max, 10th, and min (1000th) scores are summarised and the INP and AFP faces predicted to interact are displayed based on the top 10 docking arrangements (**below**).

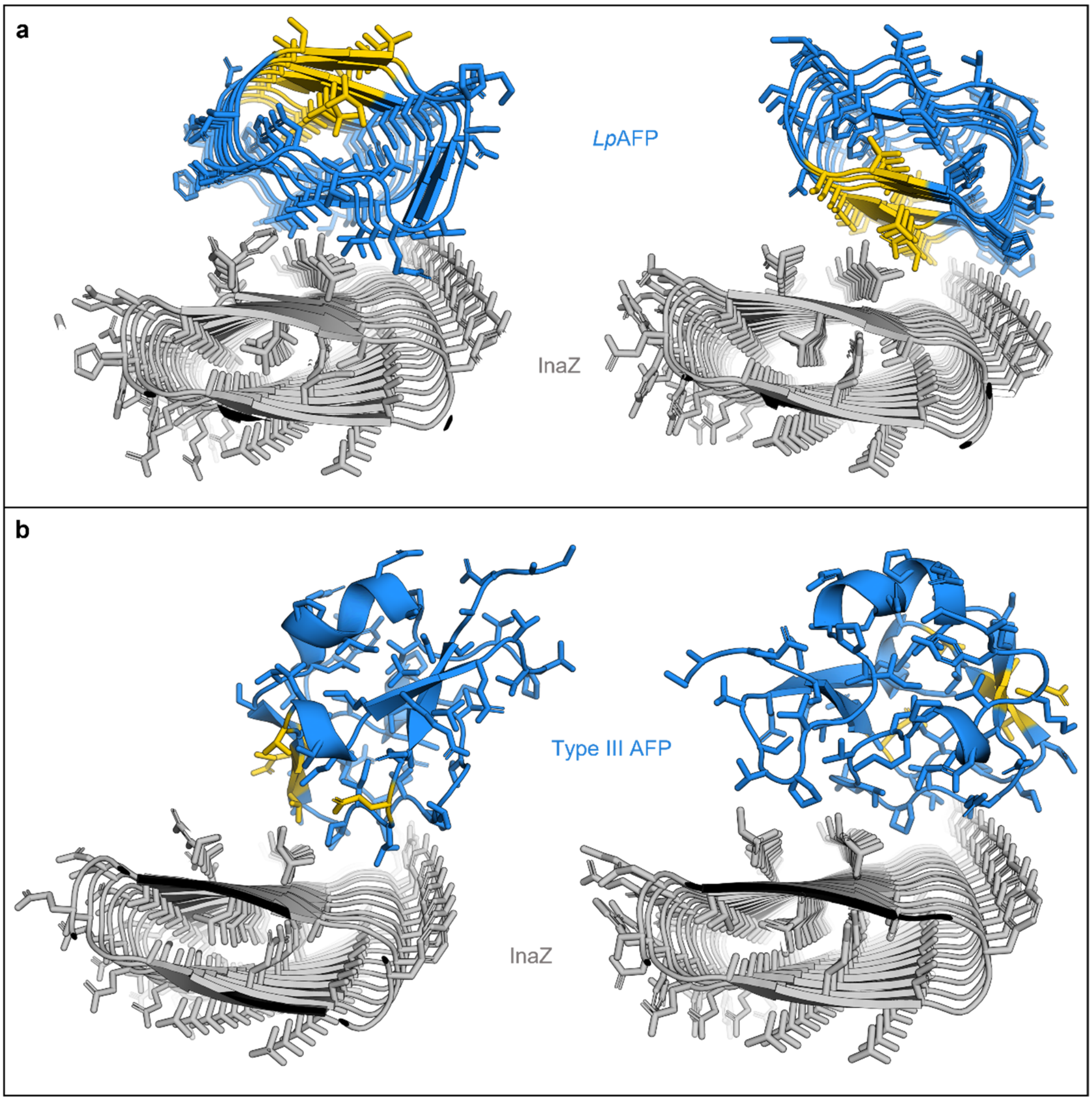

Figure S6. Cross sections of FRODOCK docking predictions of antifreeze proteins (AFPs) in blue with their ice-binding residues in yellow, on the AlphaFold model of the *Pseudomonas syringae* ice nucleating protein (INP) in grey. Note that AFPs were predicted to bind along the entire length of the INP solenoid and only the top representative images shown. (**a**) *Lolium perenne* AFP, *Lp*AFP, crystal structure model (PDB ID: 3ULT). Only the "a-side" of the *Lp*AFP has ice-binding affinity [27]. The FRODOCK algorithm suggests that docking could occur on either face, thus two images shown involve the b-face (**left** as seen in 80% of the top models) or the opposite ice-binding a-face (**right**) seen in 20% of the models. (**b**) Type III AFP from *Macrozoarces americanus* crystal structure model (PDB ID:1MSI). Shown are two representative images, one showing docking that involves the ice-binding face (**left**, seen 50% of the time) and the other elsewhere (**right**). Type III AFP does not attenuate bacterial ice nucleation [28] and the docking scores are lower than those shown by *Bd*AFP or *Lp*AFP as stated in (see Results with details provided in Figure S5).

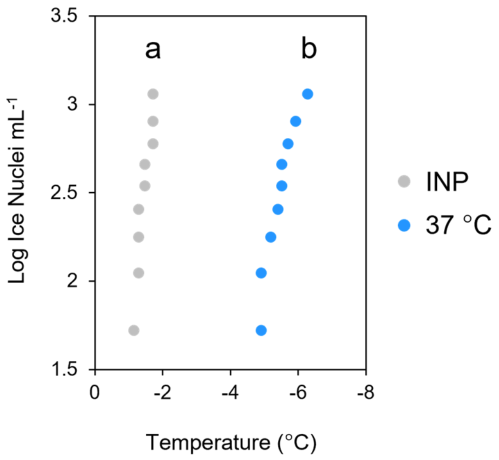

Figure S7. Ice nucleation assay comparing *P. syringae* ice nucleating protein (INP) preparations alone (in grey) to INPs maintained at 37 °C for 24 h (in blue) before assessment. Assay was repeated in triplicate with similar results. Small letter groupings indicating significance ($p < 0.001$, one way ANOVA) are shown. Samples (10 per plate) were repeated in triplicate with similar results.

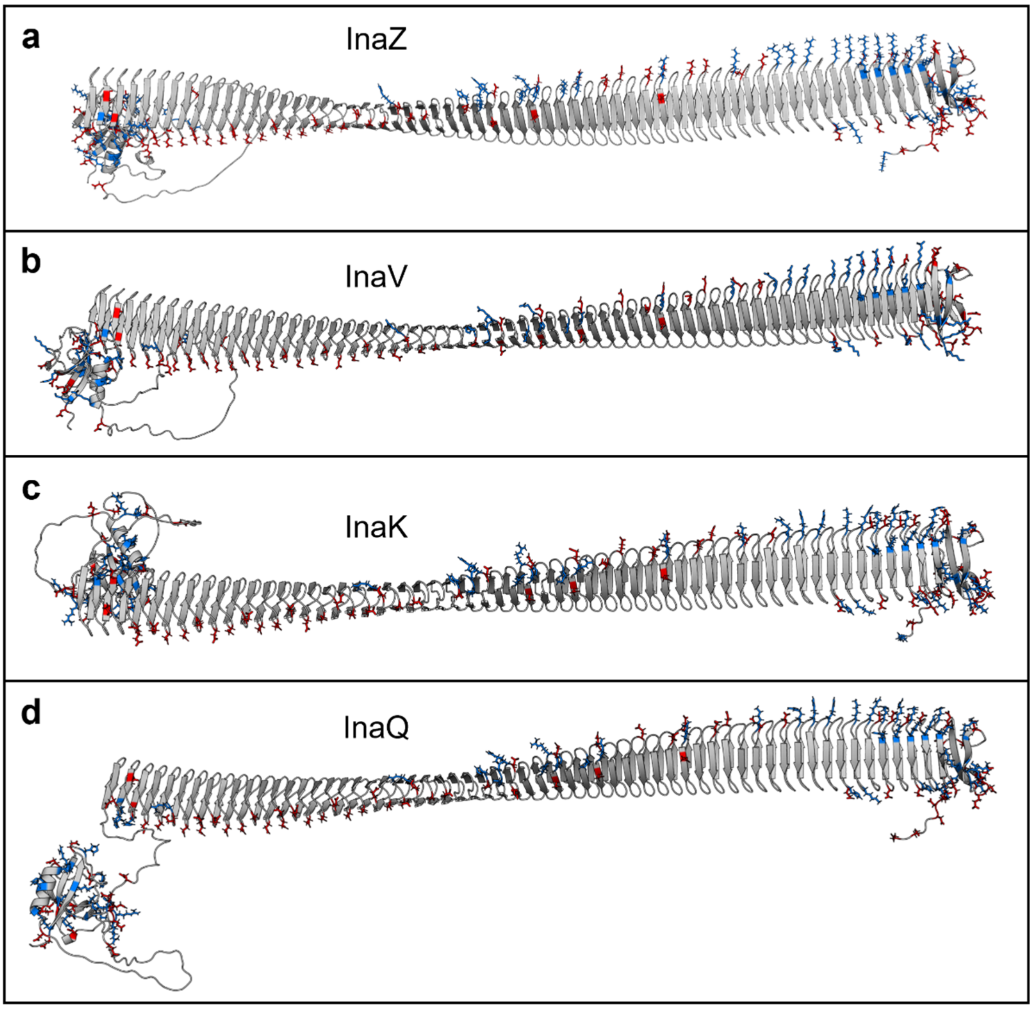

Figure S8. AlphaFold models highlighting charged residues of *Pseudomonas syringae* ice nucleating protein (INP) variants. (**a**) The INP used as the model for this work, InaZ. (**b**) variant InaV. (**c**) variant InaK. (**d**) variant InaQ. Negatively charged residues are highlighted in red and positively charged residues in blue. We propose that these positively and negatively charged residues may be involved in stabilising the formation of INP aggregate sheets by INP filaments formed through tyrosine ladder interactions.

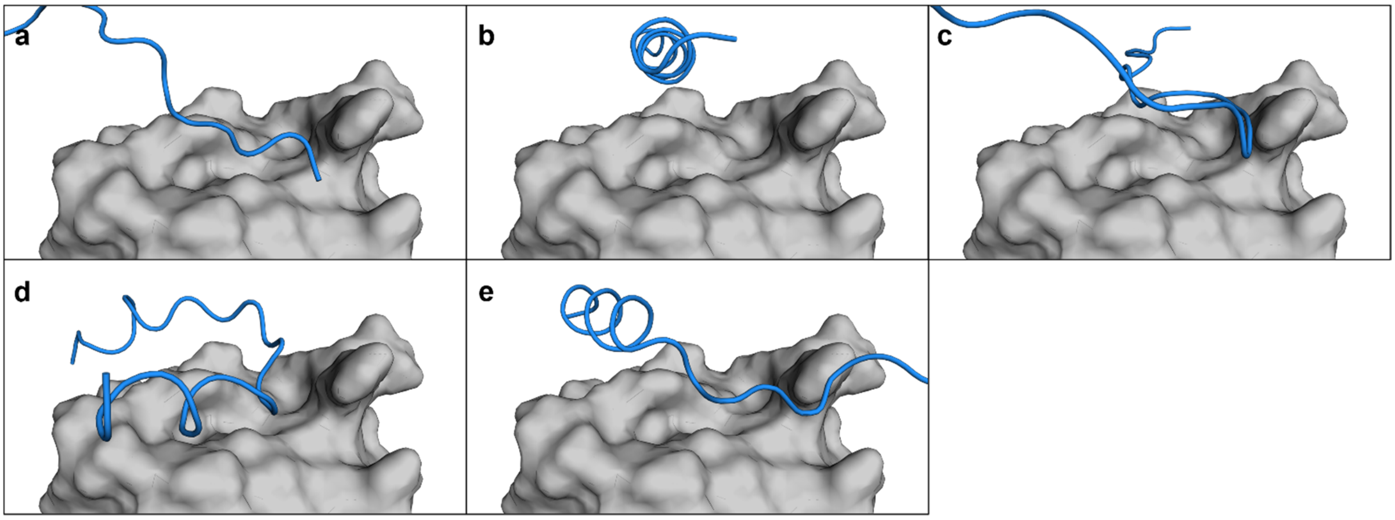

Figure S9. Gamma flg22 epitope permutation binding to the LRR corresponding to *BdIRI1*. (**a**) Close up of the flg22 predicted binding site on LRR1 (from *BdIRI1*) with the gamma flg22 permutation #1; (**b**) permutation #2; (**c**) permutation #3; (**d**) permutation #4; and (**e**) permutation #5. LRRs are represented in grey and gamma flg22 permutation peptides are in blue. No hydrogen bonds were predicted between the LRR and any epitope permutations.

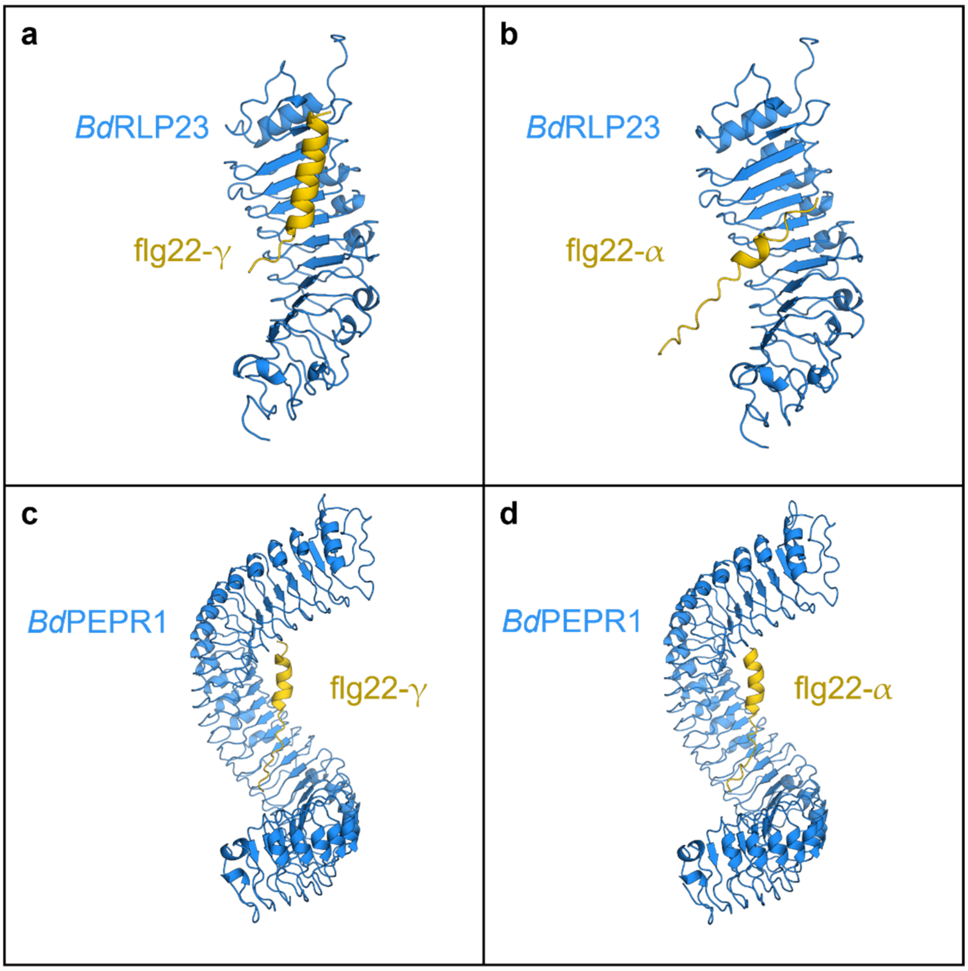

Figure S10. Control non-flg22 LRR binding proteins modelled using AlphaFold with flg22 epitopes showing reduced favourable binding affinities between receptor proteins and ligand flg22 peptides suggesting stronger affinity between *BdIRI* LRR proteins and flg22-γ peptides. (**a**) *Bd*RLP23 modelled with flg22-γ. (**b**) *Bd*RLP23 modelled with flg22-α. (**c**) *Bd*PEPR1 modelled with flg22-γ. (**d**) *Bd*PEPR1 modelled with flg22-α.
